# Differential chemosensitivity to antifolate drugs between RAS and BRAF melanoma cells

**DOI:** 10.1186/1476-4598-13-154

**Published:** 2014-06-19

**Authors:** Imanol Arozarena, Ibai Goicoechea, Oihane Erice, Jennnifer Ferguson, Geoffrey P Margison, Claudia Wellbrock

**Affiliations:** 1 Manchester Cancer Research Centre, The University of Manchester, Michael Smith Building, Oxford Road, Manchester M13 9PT, UK; 2 Oncology area, Biodonostia Research Institute, Calle Doctor Begiristain, San Sebastian 20014, Spain; 3 Division of Hepatology and Gastroenterology, Biodonostia Research Institute, Calle Doctor Begiristain, San Sebastian 20014, Spain; 4 Centre for Occupational and Environmental Health, The University of Manchester, Stopford Building, Oxford Road, Manchester M13 9PL, UK

**Keywords:** Melanoma, RAS, BRAF, Alkylating agents, DNA synthesis, Salvage

## Abstract

**Background:**

The importance of the genetic background of cancer cells for the individual susceptibility to cancer treatments is increasingly apparent. In melanoma, the existence of a *BRAF* mutation is a main predictor for successful BRAF-targeted therapy. However, despite initial successes with these therapies, patients relapse within a year and have to move on to other therapies. Moreover, patients harbouring a wild type *BRAF* gene (including 25% with *NRAS* mutations) still require alternative treatment such as chemotherapy. Multiple genetic parameters have been associated with response to chemotherapy, but despite their high frequency in melanoma nothing is known about the impact of BRAF or NRAS mutations on the response to chemotherapeutic agents.

**Methods:**

Using cell proliferation and DNA methylation assays, FACS analysis and quantitative-RT-PCR we have characterised the response of a panel of NRAS and BRAF mutant melanoma cell lines to various chemotherapy drugs, amongst them dacarbazine (DTIC) and temozolomide (TMZ) and DNA synthesis inhibitors.

**Results:**

Although both, DTIC and TMZ act as alkylating agents through the same intermediate, NRAS and BRAF mutant cells responded differentially only to DTIC. Further analysis revealed that the growth-inhibitory effects mediated by DTIC were rather due to interference with nucleotide salvaging, and that NRAS mutant melanoma cells exhibit higher activity of the nucleotide synthesis enzymes IMPDH and TK1. Importantly, the enhanced ability of RAS mutant cells to use nucleotide salvaging resulted in resistance to DHFR inhibitors.

**Conclusion:**

In summary, our data suggest that the genetic background in melanoma cells influences the response to inhibitors blocking *de novo* DNA synthesis, and that defining the RAS mutation status could be used to stratify patients for the use of antifolate drugs.

## Background

Cutaneous melanoma is a deadly form of skin cancer that develops from melanocytes, specialized pigmented cells that reside underneath the epidermis. 50% of melanomas harbour activating mutations in the kinase BRAF, the most common being a V600E substitution [1], and 25% harbour mutations in the GTPase NRAS. Both oncogenes stimulate the MAP-kinase (MAPK)-pathway, which is found hyperactivated in 90% of all melanomas [2]. Whereas BRAF only activates the MAPK-pathway, NRAS activates several other effectors including Ral-GDS or PI3-kinase, which is of special relevance for melanoma [2].

It is now accepted that genetic lesions in BRAF and NRAS have different consequences in melanoma formation and it is becoming apparent that BRAF can regulate invasion and metastasis through mechanisms different to NRAS [3].

Importantly, the genetic background of melanoma also impacts on the response to therapies targeting the MAPK-pathway. While there is no efficient targeted therapy against wild type BRAF melanomas, BRAF mutant (mutBRAF) melanomas are addicted to the MAPK-pathway and small molecule inhibitors targeting either mutBRAF or MEK have shown impressive clinical responses [4-7]. Unfortunately, these responses are transient, and patient relapse due to acquired resistance [8]. In contrast to mutBRAF melanomas, mutNRAS tumours are largely resistant to BRAF inhibitors [9,10], and moreover these drugs paradoxically stimulate the MAPK-pathway [11]. Thus, despite the initial successes with BRAF targeted therapy, relapsed patients as well as the 50% of patients harbouring a wild type BRAF (including the 25% with NRAS mutations) will still require alternative treatment such as chemotherapy and/or immunotherapy.

In melanoma some of the most commonly used chemo-therapeutics are the monofunctional-alkylating agents dacarbazine (DTIC) and temozolomide (TMZ), the chloro-ethylating agents carmustine or the bifunctional alkylating agents like cisplatin [12]. Also anti-mitotic drugs like paclitaxel and vinblastine are used in the treatment of melanoma patients (http://www.cruk.org). Historically the prodrug DTIC has been the first line treament with an average overall response of 20% [12]. In patients DTIC is metabolized in the microsomes of hepatocytes into MTIC, which undergoes spontaneous transformation into a toxic DNA methylating agent [13]. More recently patients are being treated with Temozolomide (TMZ), which does not require metabolic activation, but spontaneously converts into MTIC and shows a clinical response almost identical to DTIC [13,14].

Notably, compared to brain tumors, melanoma responses to alkylating agents are poor. In patients with malignant melanoma, the overall response to temozolomide is around 15% compared to 47 and 61% in glioma and astrocytoma patients [12,15]. Interestingly mutBRAF melanoma patients have shown responses to DTIC of up to 23% [16] but, on the other hand, recent reports state that activating mutations in BRAF have no impact on the response of stage IV melanoma patients to DTIC or TMZ [17]. However, BRAF and NRAS mutation status has never been tested retrospectively for its potential as predictive marker for DTIC responses. Resistance to these alkylating agents is thought to be due to several factors, including the altered expression of components of the apoptotic and DNA damage repair machineries and to multi-drug resistance phenotype-associated proteins such as the ABC drug transporters [18-20]. Pre-clinical studies using melanoma and glioma cells and xenografts have shown that expression of the DNA repair protein *O*^6^-methylguanine-DNA-methyl-transferase (MGMT) confers resistance to mono-alkylating agents such as DTIC, TMZ and carmustine [21]. However, this has not been successfully translated into the clinic and the use of MGMT inactivating agents to sensitise cancer cells to alkylating drugs has not provided any clinical benefit [21]. Thus, in contrast to targeted therapy, and despite extensive studies into DNA repair mechanisms in relation to tumour response, there are no good markers to predict a patient’s response to chemotherapy. Since in melanoma the genetic background delineates specific mechanisms of proliferation, survival or invasion/migration and regulates the response of melanoma cells to targeted therapy, we hypothesized with the possibility that mutations in BRAF or NRAS might affect melanoma cell response to chemotherapeutic agents.

## Results

### NRAS mutant melanoma cells are less responsive to DTIC than BRAF mutant cells

**Figure 1:**
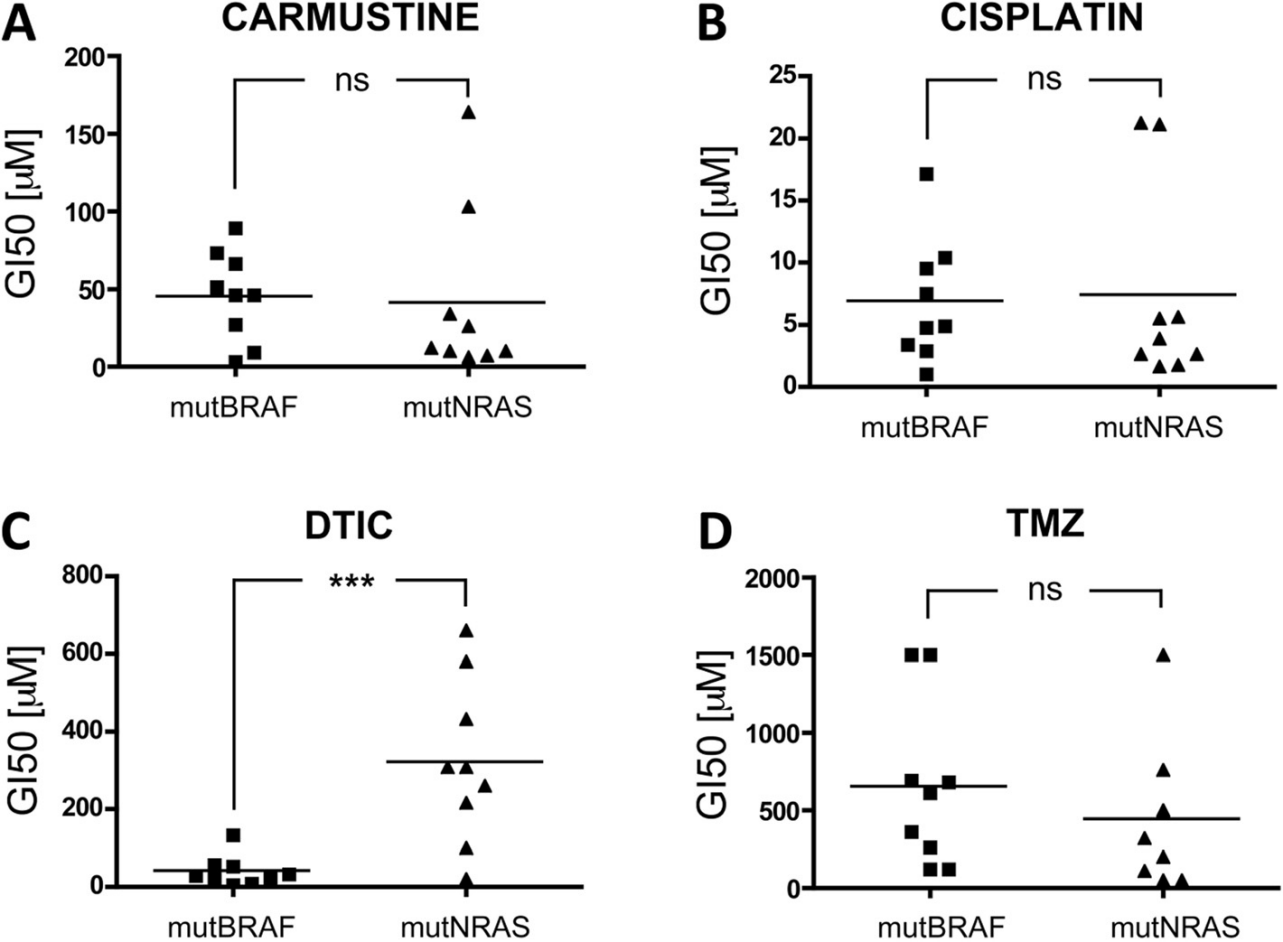
**Mutation dependent response to alkylating agents.** Nine melanoma cell lines with activating mutations in either BRAF or RAS were treated with serial increasing concentrations of **A**, carmustine **B**, cisplatin, **C**, dacarbazine (DTIC) or **D**, temozolomide (TMZ). After 5 days, cells were quantified using toluidine blue staining. Untreated cells were set 100% and the GI50 for each drug was calculated as the mean of 3 independent measurements. Each point corresponds to an individual cell line. Student’s t test compares the average GI50 for mutBRAF cell lines vs mutNRAS cell lines. ns = not significant, ***p = 0.0006 for DTIC MutBRAF vs mutRAS cells.

To address the potential influence of the genetic background on the response of melanoma cells to chemotherapeutic agents, we tested three different classes of DNA damaging agents: carmustine, cisplatin and DTIC in 9 NRAS mutant and 9 BRAF mutant melanoma cell lines (mutNRAS and mutBRAF cells; see Additional file 1: Table S1). When comparing the mean GI50 for all mutNRAS cells with that of mutBRAF cells, no statistically significant difference was observed when cells were treated with carmustine or cisplatin (Figure 1A-B). In contrast, mutNRAS cells were significantly more resistant to DTIC than mutBRAF cells (p < 0.001, Figure 1C). In view of this result we decided to use TMZ as another triazene that acts on DNA through a mechanism identical to that of DTIC. Surprisingly, no significant difference was detected between the average GI50 for TMZ in mutBRAF and mutNRAS melanoma cells (Figure 1D).

### Light activated DTIC does not act through DNA alkylation

**Figure 2:**
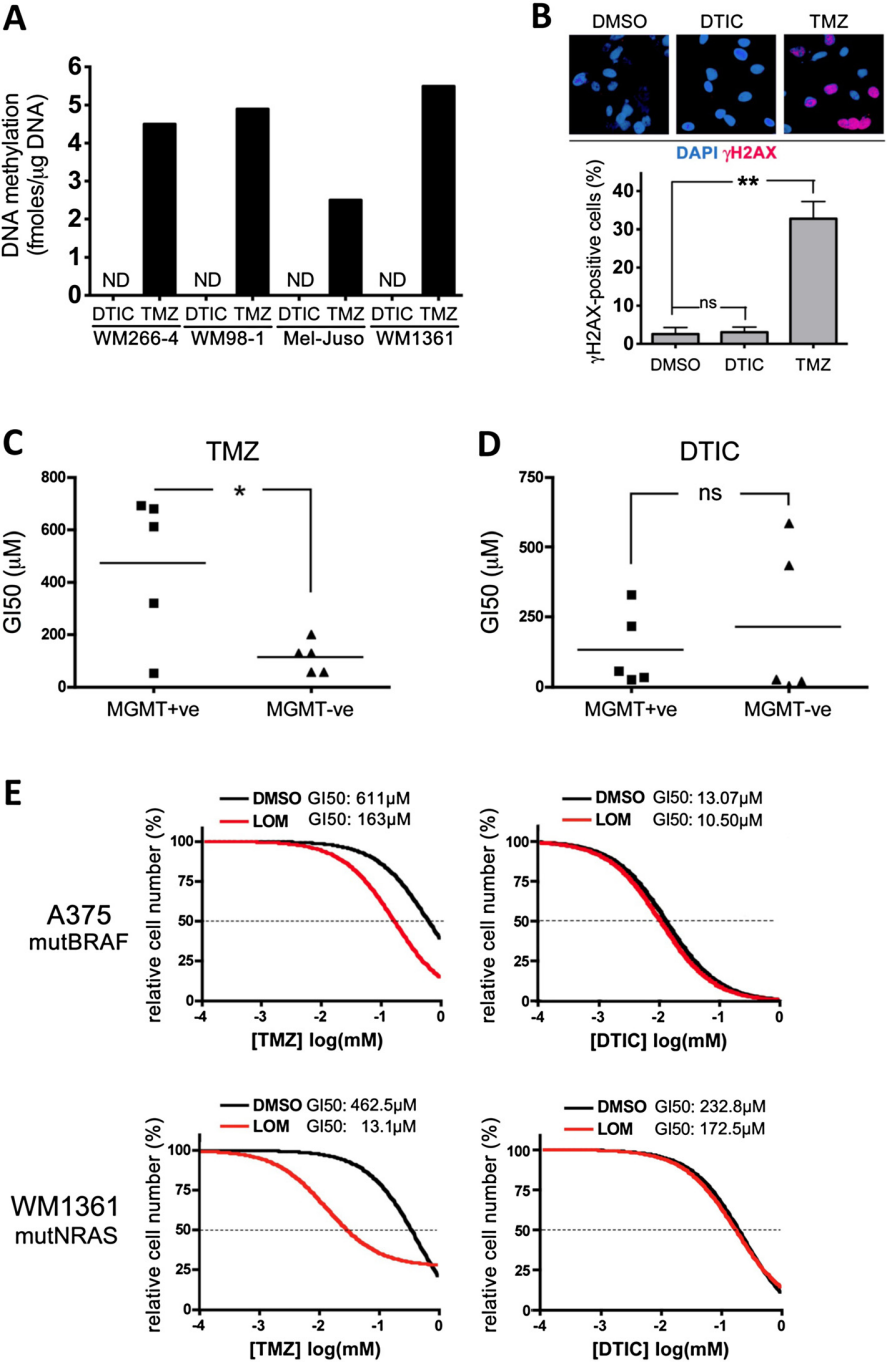
**Light activated DTIC does not methylate DNA. A**, Cells were treated with 300 μM DTIC for 24 h or 1 mM TMZ for 3 h, cells were harvested, genomic DNA purified and equal amounts of gDNA were used to determine the level of DNA methylation. ND: not detectable. **B**, Cells were treated with 300 μM DTIC or 1 mM TMZ for 24 h and stained for γH2AX by immunofluorescence. **C** and **D**, Cell lines were grouped upon their MGMT status (MGMT + ve = MGMT proficient; MGMT-ve = MGMT deficient), and the average GI50 for TMZ or DTIC was compared between both groups. Student’s t test compares the average GI50 for MGMT + ve cells vs MGMT-ve cell. n.s.: not significant, *p < 0.05. MGMT + ve cells are significantly more resistant to TMZ than MGMT-ve cells, p = 0.0116. **E**, Dose response curve of cell survival to TMZ or DTIC in the absence (DMSO) or presence of the MGMT inhibitor lomeguatrib (LOM, 20 μM). A375 or WM1361 cells were treated with lomeguatrib 1 h before addition of different concentrations of DTIC or TMZ. After 5 days cells were stained with toluidine blue and quantified. DMSO treated cells were set as 100%. The GI50 for each combinatorial treatment is shown.

As mentioned TMZ undergoes spontaneous activation, whereas DTIC needs to be metabolized in the liver [13]. We had activated DTIC by exposure to white light, an alternative *in vitro* activation method previously described by others. Indeed we confirmed that light activation enhanced DTIC-mediated growth inhibition (Additional file 2: Figure S1A). To establish that this gives rise to a DNA alkylating agent, we quantified *O*^6^meG levels in DNA extracted from DTIC-treated cells and compared them with the levels in TMZ-treated cells. As expected, TMZ efficiently induced DNA methylation (Figure 2A). However, light activated DTIC even at high concentrations (300 μM), was unable to induce any detectable DNA methylation in either mutBRAF or mutNRAS cell lines (Figure 2A). Furthermore, in line with the induction of DNA damage, Histone H2AX phosphorylation was detectable in TMZ treated cells, but no signal was observed in DTIC treated cells (Figure 2B). Accordingly, light activated DTIC failed to induce the activation of CHK-1 and CHK-2, kinases known to be activated by DNA damage (Additional file 2: Figure S1B).

Because MGMT activity is closely linked to MTIC mediated DNA damage, we determined the levels of MGMT in selected cell lines. We found no correlation between MGMT activity and NRAS or BRAF mutation status (Additional file 3: Table S2). However, as expected, MGMT expressing cells were significantly more resistant to TMZ (Figure 2C), but the average GI50 for DTIC was not significantly different between MGMT expressing and non-expressing cells (Figure 2D). Furthermore, A375P cells, which possess MGMT activity (Additional file 3: Table S2), are sensitized to TMZ by the MGMT inhibitor lomeguatrib, whereas lomeguatrib did not have any effect on DTIC treatment (Figure 2E). Similar results were obtained with other mutBRAF cells as well as with several mutNRAS cell lines including WM1361 (Figure 2E). These data indicate that light-activated DTIC, unlike TMZ, cannot induce DNA alkylation, and that the differential growth inhibitory activities observed in BRAF and NRAS mutant melanoma cells are due to alternative mechanisms.

### Light activated DTIC induces a G1 cell cycle arrest

**Figure 3:**
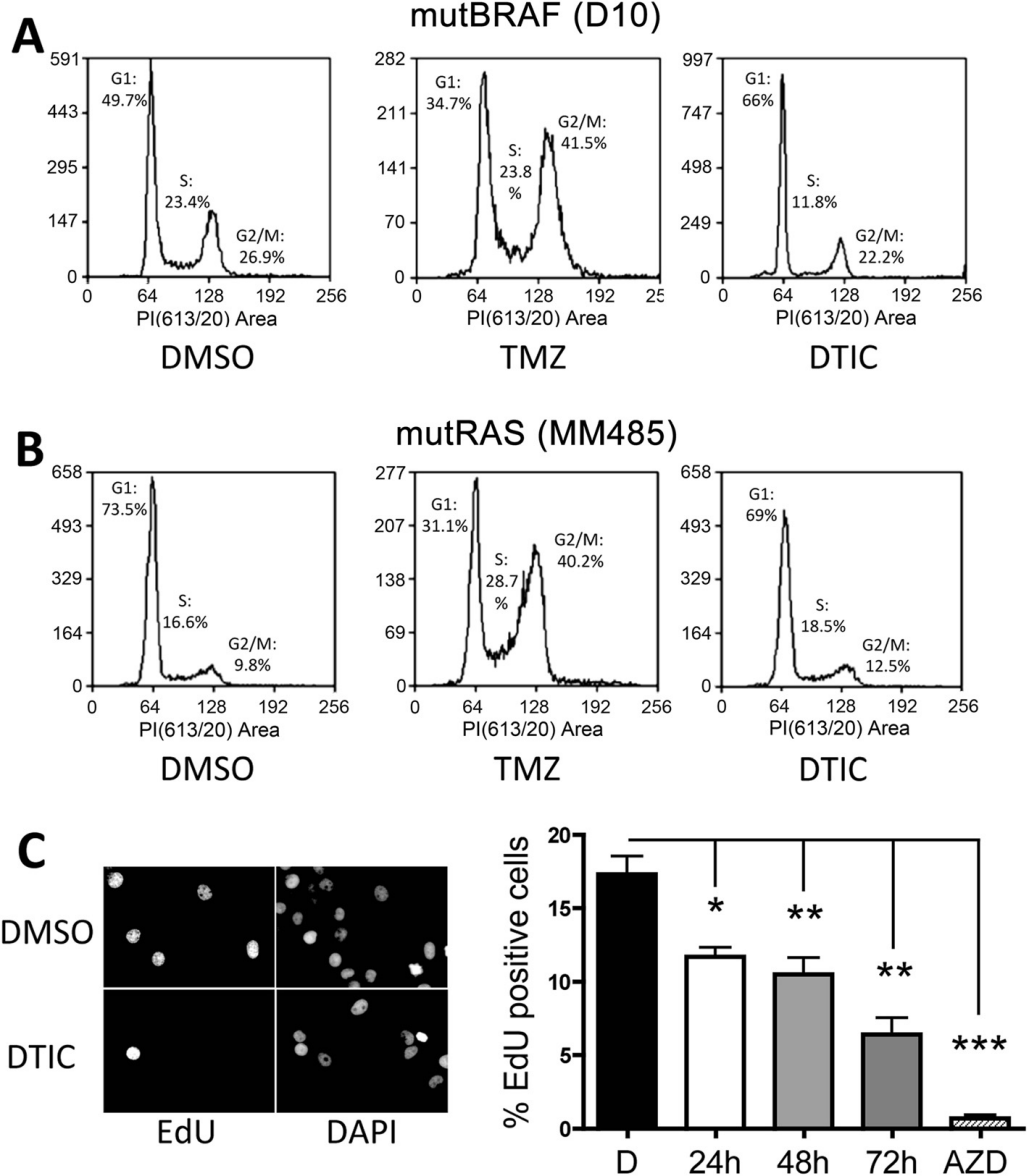
**Light activated DTIC blocks melanoma cell proliferation. A** and **B**, DNA content FACS analysis of D10 and MM485 cell lines treated with TMZ or DTIC for 72 hr. **C**, Proliferation assay. WM266-4 cells were treated with DTIC or the MEK inhibitor selumetinib and EdU incorporation was measured 24, 48 or 72 hours after drug addition as indicated; untreated cells were set 100%. One-way Anova was used to compare the effect of each treatment with untreated cells. *p < 0.05, **p < 0.01, ***p < 0.001.

To gain more insight into the effect of DTIC on melanoma cell growth we analysed cell cycle progression in the presence of light activated DTIC and compared this to TMZ treatment. Exposure to TMZ for 72 hours led to a significant G2/M arrest in both mutBRAF D10 cells and mutNRAS MM485 cells (Figure 3A and B). On the other hand, DTIC treatment with a concentration equivalent to the average GIC50 of all mutBRAF cells (50 μM) led to block at the G1/S-transition in mutBRAF D10 cells (Figure 3A). Moreover, mutNRAS MM485 cells were largely unaffected by DTIC (Figure 3B). Similar results where obtained with other mutBRAF and mutNRAS melanoma cell lines (data not shown). We then analysed whether the accumulation in G1 was linked to a reduction in DNA synthesis and indeed, when we treated mutBRAF WM266-4 cells with DTIC for 24, 48 or 72 h, there was a progressive reduction in DNA synthesis, with a maximal inhibition of 65% at 72 h (Figure 3C).

### Hypoxanthine overcomes the DTIC mediated growth inhibition in melanoma cells

**Figure 4:**
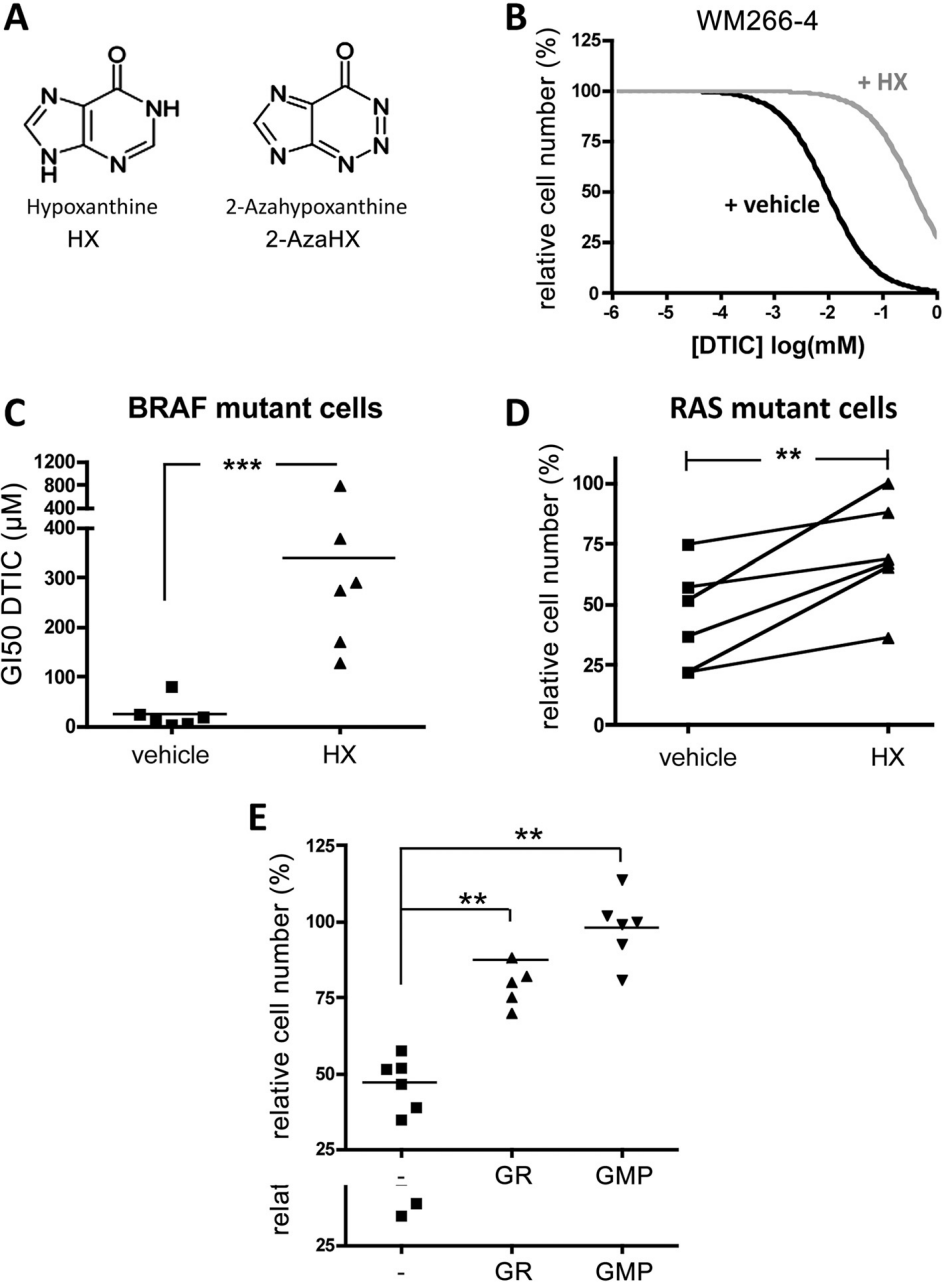
**The DTIC inhibitory effect is overcome by hypoxanthine. A**, Structure of hypoxanthine (HX) and 2-azahypoxanthine (2-AzaHX). **B**, Dose response curve of WM266-4 cell survival to DTIC in the absence (vehicle) or presence of 100 μM hypoxanthine (HX). Untreated cells were set as 100%. **C**, Graph showing the GI50 for DTIC of six mutBRAF melanoma cell lines treated as in B. The average GI50 for cells co-treated with HX (336.5 μM) was significantly higher than for vehicle-treated cells (22.89 μM), as determined by Student’s t test. **p = 0.0046. **D**, Quantification of cell survival of five mutRAS melanoma cell lines upon treatment with 300 μM DTIC in the absence (vehicle) or presence of 100 μM hypoxanthine (HX). **E**, Cell survival of 3 mutBRAF and 3 mutNRAS cell lines treated with 50 μM or 300 μM DTIC respectively in the absence (-) or presence of 100 μM Guanosine (GR) or 2′5′-GMP (GMP).

The observation of a DNA synthesis phenotype was intriguing, because white light exposure of DTIC triggers its degradation to 2-aza-hypoxanthine (2-AzaHX) [22], which can interfere with DNA synthesis. Thereby 2-AzaHX competes with the structurally related hypoxanthine (HX) as substrate for the hypoxanthine-guanine phosphoribosyl–transferase (HGPRT) in the purine salvage pathway (Figure 4A).

To assess the possibility that DTIC was transformed into 2-AzaHX, we analysed the UV absorption spectrum of DTIC and observed that light exposure of DTIC led to the formation of a metabolite with maximal UV absorption at 256-295 nm, similar to that of 2-AzaHX (Additional file 4: Figure S2).Next we treated the melanoma cell lines with DTIC in the presence of HX, which should compete with 2-AzaHX for HGPRT binding and prevent its inhibitory effect. Indeed, the addition of HX significantly overcame the effects of DTIC in WM266-4 cells, increasing the GI50 to DTIC by almost 15-fold (Figure 4B). Moreover, in the presence of HX, the average GI50 for DTIC in mutBRAF cells was comparable to that of mutNRAS melanoma cells in the absence of HX (~300 μM, Figure 4C). Notably, when mutNRAS melanoma cells were treated with 300 μM DTIC HX addition also reverted the growth effects (Figure 4D), suggesting that DTIC inhibits the purine salvage pathway also in these cells. In support of this finding, the addition of 5′-guanosine monophosphate (GMP) or guanosine (GR) rescued the inhibitory effect of DTIC in mutBRAF and mutNRAS cells (Figure 4E). These results support the hypothesis that the growth inhibitory effect of light activated DTIC is mediated by its degradation to 2-AzaHX, which inhibits the purine salvage pathway.

### NRAS mutant melanoma cells display higher nucleotide salvage pathway activity than BRAF mutant cells

**Figure 5:**
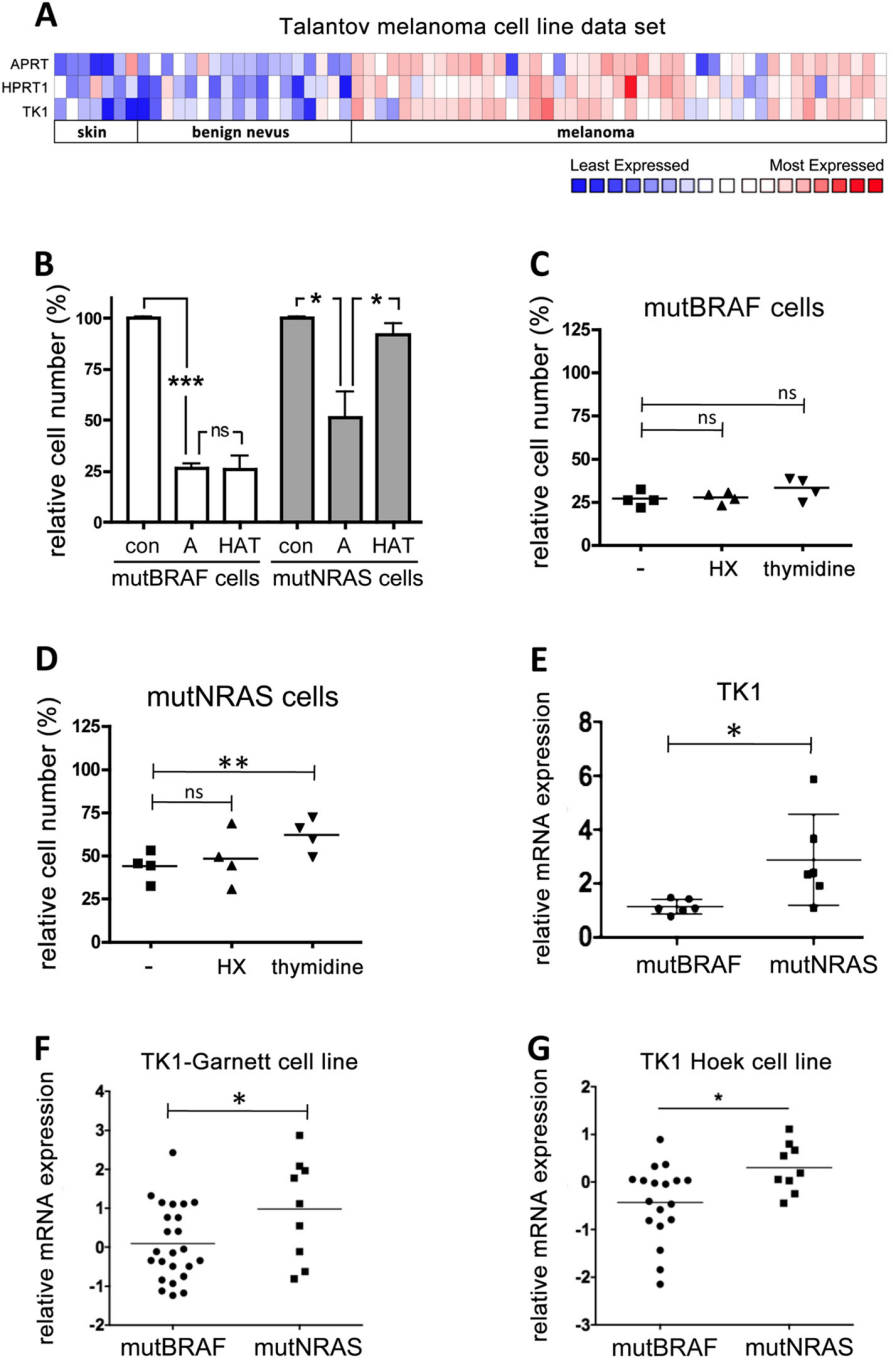
**mutNRAS melanoma cells possess increased thymidine salvage capacity. A**, Heat map of expression profile of APRT, HPRT1 and TK1 genes in normal skin, benign nevus and melanoma in a data set obtained from Oncomine [24]. **B**, Four mutBRAF and mutNRAS melanoma cell lines were treated with 0.4 μM aminopterine in the absence (A) or presence of hypoxanthine and thymidine (HAT). After 3 days cells were fixed, stained with toluidine blue and surviving fractions were quantified. **C**, Four mutBRAF or **D**, mutNRAS cell lines were grown in normal medium supplemented with 0.4 μM aminopterin in the presence or absence of 100 μM HX or 16 μM thymidine, as indicated. After 3 days the survival fraction was determined. Cells cultured in normal medium were set as 100% survival. **E-G**, Comparison of thymidine kinase (TK1) mRNA expression in mutBRAF and mutNRAS melanoma cell lines (as assessed by q-RT-PCR) in our panel of melanoma cell lines or in two independent data sets deposited in Oncomine [25,26]. *p < 0.05, **p < 0.01, ***p < 0.001.

Nucleotide salvage pathways are crucial for efficient DNA synthesis, particularly in fast dividing cells. In line with this, we found increased expression of the key enzymes of nucleotide salvaging HGPRT (HPRT1), thymidine kinase (TK1) and APRT in melanoma (primary and metastatic) compared to normal skin and benign nevi ( Figure 5A). We then argued that despite the general increased salvage activities in melanoma cells, mutNRAS cells might exhibit and even higher efficiency of salvage pathway usage than mutBRAF cells, which would render them resistant to the inhibitory effect of 2-AzaHX. To test this hypothesis we cultured melanoma cell lines in the presence of an inhibitor of *de novo* DNA synthesis, aminopterin. Under these conditions cell growth is mainly driven via nucleotide salvage pathways, which is fuelled by the addition of the supplements HX and thymidine 005B [23]. In the presence of aminopterin, the growth of all cell lines was significantly reduced (Figure 5B), indicating that de novo DNA synthesis is required for cell growth. However, whereas the addition of HX and thymidine almost completely rescued the growth of mutNRAS cell lines, mutBRAF cell lines did not show an increase in cell growth (Figure 5B). This suggested that although mutBRAF cells use salvage pathways for cell growth when de novo synthesis is inhibited (25% cell growth after 3 days of inhibition), the efficiency of this alternative DNA synthesis route is much lower in these cells than in mutNRAS cells.

We next quantified the individual effects of adding HX and thymidine as salvage substrates for HGPRT and thymidine kinase, respectively. Interestingly, when the de novo synthesis was inhibited addition of HX alone did not enhance cell growth in mutNRAS and mutBRAF cells (Figure 5C and D), suggesting that under these conditions the cells might be using endogenously produced guanine as an alternative substrate [27]. On the other hand, mutNRAS cells were significantly more efficient than mutBRAF cells in utilising thymidine to counteract the growth inhibitory effect of de novo synthesis inhibition (Figure 5C and D).

Thymidine is the substrate of TK1 in the pyrimidine salvage pathway and our data suggested that TK1 activity is increased in mutNRAS cells. Indeed, we found a significant overexpression of TK1 in mutNRAS cells compared to mutBRAF cells in our panel of melanoma cell lines (Figure 5E). This finding was supported by two independent datasets [25,26] analysed in Oncomine (Figure 5F and G).

### NRAS mutant melanoma cells are more resistant to DNA *de novo* synthesis inhibitors than BRAF mutant cells

**Figure 6:**
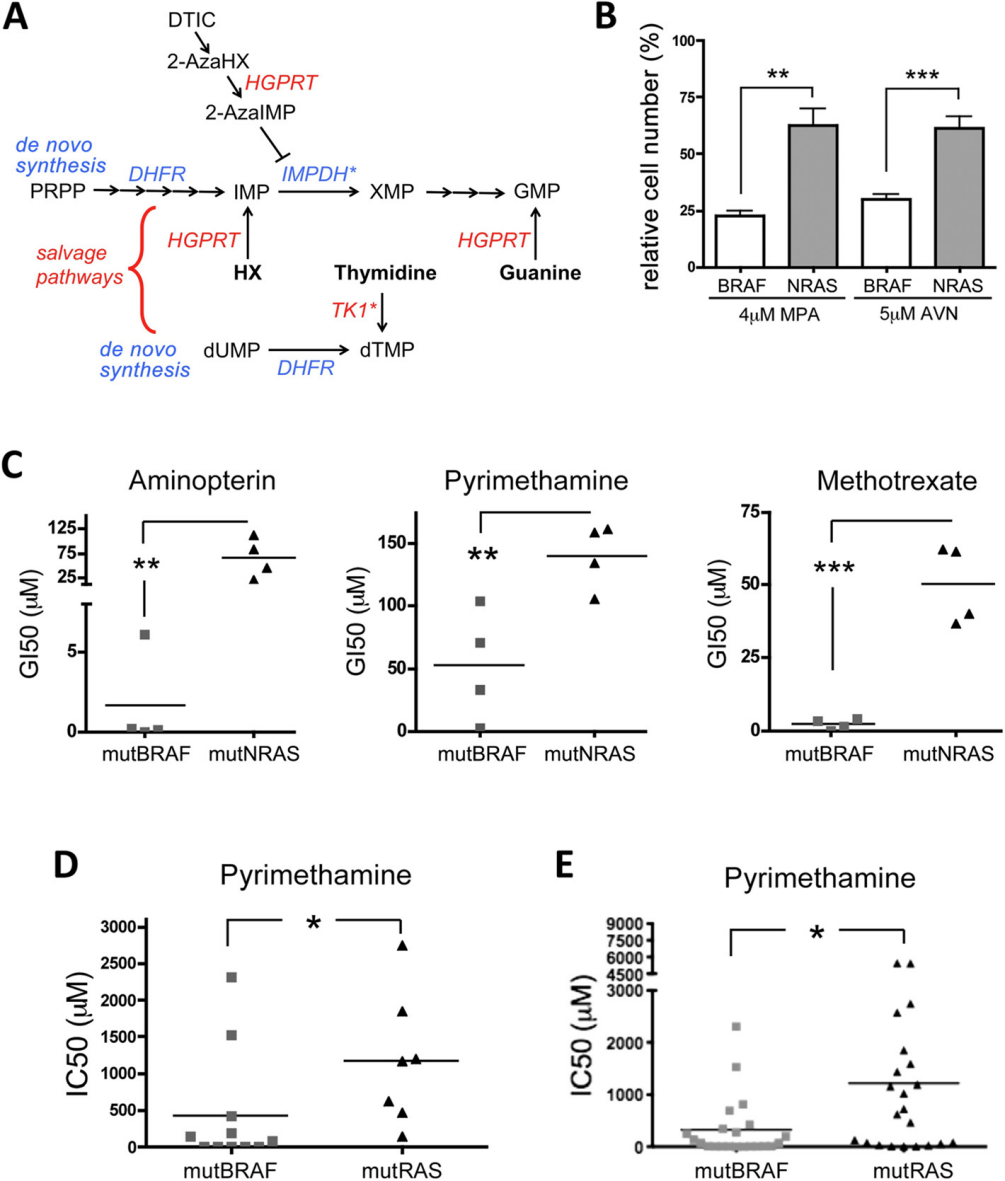
**mutRAS melanoma cells are more resistant to DHFR targeting drugs. A**, Schematic of nucleotide de novo synthesis and salvage pathways. The asterisks indicate increased activity (IMPDH) or expression (TK1) in mutNRAS cells. **B**, Average survival of 4 mutBRAF or mutNRAS cell lines after treatment with 4 μM Mycophenolic Acid (MPA) or 5 μM AVN499. **p = 0.01, ***p < 0.001. **C**, Melanoma cell lines with activating mutations in either BRAF or NRAS were treated with serial increasing concentrations of aminopterin, pyrimethamine or methotrexate. After 3 days, cells were fixed, stained and the GI50 for each drug was calculated. Student’s t test (one-tailed) compares the average GI50 for mutBRAF cell lines vs mutNRAS cell lines. Aminopterin: **p = 0.0092; Pyrimethamine: **p = 0.0071; Methotrexate: ***p = 0.0002. **D**, Comparison of the IC50 for dypirimethamine of 11 mutBRAF and 7 mutRAS melanoma cell lines, as determined by MTS assay in [25]. *p = 0.0393. **E**, Graph comparing the IC50 for dypirimethamine between 22 mutBRAF and 22 mutNRAS cell lines from different tumor types [25]. Student’s t test, two-tailed: *p = 0.0184.

Our data show that in mutNRAS melanoma cells elevated TK1 activity contributes to enhanced pyrimidine salvaging. However, the inhibitory effect of 2-AzaHX is on the purine salvage pathway, where after conversion into 2-Aza-inositol monophosphate (2-AzaIMP) it suppresses IMP dehydrogenase (IMPDH) (Figure 6A). Thus, the difference in the response of mutNRAS and mutBRAF cells to DTIC could be based on differences in IMPDH. Indeed, mutNRAS cells were significantly more resistant to two IMPDH inhibitors, Mycophenolic Acid (MPA) and AVN944, compared to mutBRAF cells (Figure 6B). IMPDH expression levels did not differ in the individual cell lines (data not shown), indicating that the resistance in mutNRAS cells is not due to higher IMPDH expression levels.

In summary, mutNRAS melanoma cells are more efficient in nucleotide salvaging than mutBRAF melanoma cells, which is at least part due to enhanced *TK1* expression and IMPDH activity (Figure 6A). This finding suggests that mutNRAS melanoma cells would be more resistant to drugs targeting DNA *de novo* synthesis than mutBRAF cells. Indeed, when we determined the GI50 of our panel of melanoma cells for the DHFR inhibitors aminopterin, pyrimethamine and methrotrexate, we found that mutNRAS cells were significantly more resistant than mutBRAF cells (Figure 6C). Moreover, when we analysed a dataset derived from a drug screen using a large panel of cancer cell lines [25], we found not only that NRAS mutant melanoma cell lines were more resistant to pyrimethamine than BRAF mutant melanoma cells (Figure 6D), but that independently of cancer type, mutRAS cancer cells were significantly more resistant to pyrimethamine than mutBRAF cells (Figure 6E).

## Discussion

The aim of this study was to determine whether the mutational status of melanoma cells would correlate with their response to chemotherapeutic agents. Our results demonstrate that in melanoma the presence of mutually exclusive BRAF and NRAS mutations has no influence on the response to DNA alkylating agents such as TMZ. Similar results considering BRAF or RAS mutation status are found in a large data set containing drug-treatment data from 732 cancer cell lines of different origin [25]. Thus, it appears that mutBRAF and mutNRAS share common mechanisms of resistance to methylating agents such as drug efflux, or deregulation of pro-apoptotic or DNA repair pathways.

Surprisingly, we found that mutBRAF and mutNRAS cells respond very differently to light activated DTIC. Activation of DTIC by exposure to white light has been described to recapitulate its chemotherapeutic activity *in vitro*[28,29] but our results provide experimental evidence that the toxic effect described for light activated DTIC is independent of DNA methylation. Through DNA alkylation assays and combinatorial treatments using DTIC and a MGMT inhibitor we provide clear evidence that light exposure does not transform DTIC into a DNA methylating agent, but rather an inhibitor of DNA synthesis. This finding is of major importance considering that light activation of DTIC has been extensively used to study the mechanisms underlying its cytotoxic effects as well as leading to acquired resistance in patients [28-31]. In this context it is important to mention that wild type BRAF melanoma cells that had been selected for resistance to light activated DTIC *in vitro* exhibited increased tumour growth *in vivo*, a phenotype that correlates well with enhanced DNA synthesis activity [32]. Most strikingly these resistant cells and tumours displayed hyper-activation of the MAP-kinase pathway, resulting in increased IL8 and VEGF expression [28,32]. The fact that we now show that light activated DTIC inhibits nucleotide synthesis, most probably by inhibiting IMPDH, suggests a novel link between DNA synthesis pathways and MAP-kinase signalling.

Our results indicate that non-metabolically activated DTIC mediates its effects through 2-AzaHX. Strikingly, there is evidence that metabolic activation of DTIC in patients is inefficient and that, shortly after DTIC isolated limb perfusion, significant amounts of 2-AzaHX can be detected in the bloodstream and urine of patients [33]. Thus it is possible that 2-AzaHX could contribute to the DTIC-dependent toxicity, although it is well established that the anti-tumour activity of DTIC is mainly the result of DNA methylation [13].

In this context it is important to mention that conversion of 2AzaHX by HGPRT to 2-AzaIMP is able to inhibit IMPDH [34,35]. We found that mutNRAS melanoma cells are significantly more resistant to two bona fide IMPDH inhibitors (MPA and AVN944), suggesting that in NRAS mutant cells IMPDH activity or its downstream signalling is elevated. This, together with the fact that mutNRAS cells express higher levels of TK1 and consequently are more effective in using thymidine for DNA synthesis, provides strong evidence that mutNRAS melanoma cells are significantly more efficient in nucleotide salvaging.

Increased *IMPDH2* expression in cancer cells has been linked to resistance to methotrexate in osteosarcoma, colorectal and erythroleukemia cells [36-38]. However, although *IMPDH2* is overexpressed in melanoma compared to benign melanocytic lesions (not shown), its expression did not differ in mutNRAS and mutBRAF melanoma cells. Therefore, the difference in the response to IMPDH inhibitors rather suggests that IMPDH activity or its downstream signalling is regulated differently in mutNRAS compared to mutBRAF cells. Apart from IMPDH, we also show that thymidine can compensate for DHFR inhibition in resistant mutNRAS cells, which express higher levels of TK1. Whether elevated TK1 expression is directly regulated by NRAS is not yet known, but it will be crucial to identify the underlying mechanism. Importantly, we did not find differences in TK2 expression between mutNRAS and mutBRAF cells (not shown), which is maybe not surprising considering the more ubiquitous role of TK2 [39].

Historically antifolate drugs such as methotrexate or edatrexate have shown very little activity in clinical trials with melanoma patients although these trials were performed before the discovery of BRAF and RAS as drivers of melanomagenesis [40,41]. The lack of response in melanoma patients can be explained by several mechanisms of resistance such as melanosomal sequestration of drugs, the upregulation of both DHFR and the pro-survival transcription factor MITF in response to MTX, or the E2F and Chk1 mediated effects, as recently described [42-45]. Despite the inherent capacity to resist any chemotherapy our data suggest that stratifying patients according to their BRAF/RAS mutation status could lead to better responses to antifolate based therapies.

Importantly, our findings suggest that the correlation between NRAS and BRAF mutations and their differential response to antifolate drugs might apply to other cancer types. Therefore, in cancer types where antifolate based therapies contribute to achieve clinical responses in RAS patients (e.g. colorectal carcinoma) [46], it would be interesting to assess whether mutBRAF patients show even improved responses. If that were the case it would open the possibility to use mutational status as a predictor of patient response. In summary, our findings identify the mutually exclusive NRAS and BRAF mutation status as possible predictive marker for the response to DNA synthesis inhibitors such as antifolate drugs in melanoma patients.

## Conclusions

In summary in this study we demonstrate that activation of DTIC with white light does not result in a methylating agent but in to an inhibitor of purine synthesis. We show that RAS mutant melanoma cells are more resistant to drugs affecting DNA synthesis than BRAF mutant cells. Our data suggests that, the increased resistance to de novo DNA synthesis inhibitors found in RAS mutant cells is due to a superior capacity to salvage DNA. Notably our results suggest the possibility that the correlation between RAS and BRAF mutations and the response to antifolate drugs might be relevant in other cancer types although further efforts to confirm this hypothesis are warranted.

## Materials and methods

### Cell culture

Nine mutant BRAF cell lines and nine mutant NRAS cell lines were used in the study (Additional file 1: Table S1). These cells were a kind gift from Dr. Richard Marais and Dr. Adam Hurlstone. Cell stocks were expanded, frozen, and kept in liquid nitrogen. New aliquots were thawed every 5–7 weeks. Cells were cultured in Dulbecco’s Modified Eagle’s Medium (DMEM) (SIGMA) or in RPMI-164 medium (RPMI) (SIGMA) as previously indicated, supplemented with 0.5% penicillin and streptomycin (SIGMA) and 10% bovine calf serum (PAA, Yeovil, UK). Cells were grown at 37°C in a 5% CO_2_ environment.

### Reagents

HAT supplement (50X) was from Sigma. Dacarbazine, carmustine, cisplatin and temozolomide and lomeguatrib were from SIGMA. Hypoxanthine, guanosine and 5′-guanosine monophosphate were from Sigma. AZD6244 was from Selleck Chemicals, Newmarket, UK. Mycophenolic Acid and AVN944 were from Sigma and ChemieTek respectively. Aminopterin, pyrimethamine and amethopterin (methotrexate) were from Sigma. All drugs were dissolved in dimethylsulfoxide (DMSO) and, apart from dacarbazine, directly added to cell in culture at the indicated concentrations. Prior to addition onto cells DTIC was exposed to white light for 1 h, as previously described [28,30].

### Determination of MGMT activity

Melanoma cell free extracts prepared from 10^6^ cells were analysed for MGMT activity using calf thymus DNA methylated in vitro with N-nitroso-N-[^3^H]-methylurea (~20 Ci/mmol) as the substrate [47]. MGMT activity was expressed as fmol/μg DNA to avoid the possible effect of variable protein content on apparent MGMT activity expressed per unit protein [48]. No significant differences in the study results were noted when MGMT activity was expressed per unit protein. Results are the mean of quadruplicate determinations for each sample. Cell free extracts prepared from the human breast cancer cell line MCF-7 were assayed for MGMT activity as a positive control.

### Determination of O6-methylguanine levels in DNA

O6-methylguanine (*O*^6^-meG) in DNA was quantified using a modification of the standard MGMT activity assay procedure [49]. Increasing amounts of the DNA samples were pre-incubated with a standard amount of purified recombinant human MGMT [50] and residual activity was then determined. *O*^6^-meG in DNA stoichiometrically inactivates MGMT. Thus the amount of *O*^6^-meG in the DNA sample equals the amount of inactivation of the purified MGMT.

### Determination of GI50

To determine the drug concentration necessary to inhibit cell growth by 50% (GI50), 2000 cells per well were plated in 96 well plates (Corning). After 24 hours, drugs were added in triplicates in serial 1:3 dilutions. In experiments where cells were co-treated with the MGMT inactivating agent lomeguatrib, the drug was added 1 hour before the addition of serial dilutions of DTIC or TMZ. After 3 or 5 days cells were washed with PBS and simultaneously fixed and stained for 1 hour with 4% Formaldehyde (Fisher Scientific) and 0.5% Toluidine Blue (Fluka Analytical) in PBS. Plates were washed, dried and the dye was solubilized with 1% Sodium dodecyl sulfate (SDS) (Fisher Scientific) in PBS. Finally, a spectrophotometer (BIO-TEK®, NorthStar Scientific) was used to measure the O.D. and GI50 values were calculated using the GraphPad Prism software (GraphPad Software, 4.0a).

### Databases

To study the expression profile of APR1, HPRT1 and TK1 genes in human melanoma versus normal skin or benign nevus, and to compare TK1 expression between mutBRAF and mutRAS human melanoma cell lines we used Oncomine Cancer Microarray database (http://www.oncomine.org/).

### RNA isolation and qPCR analysis

RNA was isolated with TRIZOL® and selected genes were amplified by quantitative real time PCR using SYBR green (Qiagen, Valencia, CA, USA).

Primers sequences were:

TK1:

Forward: 5′-TGGCTGTCATAGGCATCGAC-3′,

Reverse: 5′-CCAGTGCAGCCACAATTACG-3′

BETA-ACTIN:

Forward: 5′-GCAAGCAGGAGTATGACGAG-3′,

Reverse: 5′-CAAATAAAGCCATGCCAATC-3′

### EdU incorporation assays

Cells were labelled with 10 μM EdU (Invitrogen) for 4 h before they were formalin fixed and processed following the manufacturer’s instructions. Stained cells were analysed using a BDpathway 855 Bioimager.

### FACS analysis

100000 cells were treated as indicated, fixed in ice-cold 80% ethanol. Cells were then washed in PBS and incubated in a solution containing PBS, RNase A and Propidium Iodide (SIGMA) at 37°C for 1 hour. The analysis was performed using FACS Calibur (Becton Dickinson).

### Statistical analysis

Unless indicated otherwise, data are from assays performed in triplicate, with error bars to represent standard deviations or errors from the mean. Statistics used were: predominately Student t-test and One-way ANOVA with Dunnett’s Multiple Comparison Test performed using GraphPad Prism version 4.00 for Mac OS, GraphPad Software, San Diego California USA, http://www.graphpad.com.

## Abbreviations

DTIC: Dacarbazine; TMZ: Temozolomide; MGMT: Methylguanine-DNA-methyl-transferase; HX: Hypoxhantine; GMP: 5′-guanosine monophosphate; GR: Guanosine; IMPDH: Inosine-5′-monophosphate dehydrogenase; HGPRT: Hypoxanthine-guanine phosphoribosyltransferase.

## Competing interests

The authors declare no conflict of interest.

## Authors’ contributions

IA conceived the study, designed and carried out most of the experiments, analysed the data and wrote the manuscript. IG and OE carried out the experiments to assess the GI50 of every alkylating agent and the effect on the cell cycle. JF was in charge of q-RT-PCR experiments. GPM carried out DNA methylation and MGMT activity assays and helped write the manuscript. CW conceived and coordinated the study, and wrote the manuscript. All authors read and approved the final manuscript.

## Supplementary Material

Additional file 1: Table S1 List of melanoma cell lines used in this study, grouped upon the presence of activating mutations in either BRAF or RAS. Click here for file

Additional file 2: Figure S1 A, DTIC exposure to white light increases DTIC’ inhibitory effect. Click here for file

Additional file 3: Table S2 MGMT status of a panel of 10 melanoma cell lines MGMT activity in 5 mutBRAF and 5 mutNRAS melanoma cell lines. Exponentially growing cells were harvested, gDNA was isolated and the presence of MGMT activity as well as the concentration of MGMT was determined. N.d.: not detectable. Click here for file

Additional file 4: Figure S2 2-Azahypoxanthine and light activated DTIC show similar UV absorbance profiles. Click here for file

## References

[B1] Davies H Bignell GR Cox C Stephens P Edkins S Clegg S Teague J Woffendin H Garnett MJ Bottomley W Davis N Dicks E Ewing R Floyd Y Gray K Hall S Hawes R Hughes J Kosmidou V Menzies A Mould C Parker A Stevens C Watt S Hooper S Wilson R Jayatilake H Gusterson BA Cooper C Shipley J Mutations of the BRAF gene in human cancer Nature 2002 417 949 954 12068308 10.1038/nature00766

[B2] Wellbrock C Hurlstone A BRAF as therapeutic target in melanoma Biochem Pharmacol 2008 80 561 567 20350535 10.1016/j.bcp.2010.03.019

[B3] Arozarena I Sanchez-Laorden B Packer L Hidalgo-Carcedo C Hayward R Viros A Sahai E Marais R Oncogenic BRAF induces melanoma cell invasion by downregulating the cGMP-specific phosphodiesterase PDE5A Cancer Cell 2011 19 45 57 21215707 10.1016/j.ccr.2010.10.029

[B4] Belden S Flaherty KT MEK and RAF inhibitors for BRAF-mutated cancers Expert Rev Mol Med 2012 14 e17 23058743 10.1017/erm.2012.11

[B5] Chapman PB Hauschild A Robert C Haanen JB Ascierto P Larkin J Dummer R Garbe C Testori A Maio M Hogg D Lorigan P Lebbe C Jouary T Schadendorf D Ribas A O’Day SJ Sosman JA Kirkwood JM Eggermont AM Dreno B Nolop K Li J Nelson B Hou J Lee RJ Flaherty KT McArthur GA BRIM-3 Study Group Improved survival with vemurafenib in melanoma with BRAF V600E mutation N Engl J Med 2011 364 2507 2516 21639808 10.1056/NEJMoa1103782 PMC3549296

[B6] Flaherty KT Infante JR Daud A Gonzalez R Kefford RF Sosman J Hamid O Schuchter L Cebon J Ibrahim N Kudchadkar R Burris HA 3rd Falchook G Algazi A Lewis K Long GV Puzanov I Lebowitz P Singh A Little S Sun P Allred A Ouellet D Kim KB Patel K Weber J Combined BRAF and MEK inhibition in melanoma with BRAF V600 mutations N Engl J Med 2012 367 1694 1703 23020132 10.1056/NEJMoa1210093 PMC3549295

[B7] Sosman JA Kim KB Schuchter L Gonzalez R Pavlick AC Weber JS McArthur GA Hutson TE Moschos SJ Flaherty KT Hersey P Kefford R Lawrence D Puzanov I Lewis KD Amaravadi RK Chmielowski B Lawrence HJ Shyr Y Ye F Li J Nolop KB Lee RJ Joe AK Ribas A Survival in BRAF V600-mutant advanced melanoma treated with vemurafenib N Engl J Med 2012 366 707 714 22356324 10.1056/NEJMoa1112302 PMC3724515

[B8] Wagle N Emery C Berger MF Davis MJ Sawyer A Pochanard P Kehoe SM Johannessen CM Macconaill LE Hahn WC Meyerson M Garraway LA Dissecting therapeutic resistance to RAF inhibition in melanoma by tumor genomic profiling J Clin Oncol 2011 29 3085 3096 21383288 10.1200/JCO.2010.33.2312 PMC3157968

[B9] Joseph EW Pratilas CA Poulikakos PI Tadi M Wang W Taylor BS Halilovic E Persaud Y Xing F Viale A Tsai J Chapman PB Bollag G Solit DB Rosen N The RAF inhibitor PLX4032 inhibits ERK signaling and tumor cell proliferation in a V600E BRAF-selective manner Proc Natl Acad Sci U S A 2010 107 14903 14908 20668238 10.1073/pnas.1008990107 PMC2930420

[B10] Solit DB Garraway LA Pratilas CA Sawai A Getz G Basso A Ye Q Lobo JM She Y Osman I Golub TR Sebolt-Leopold J Sellers WR Rosen N BRAF mutation predicts sensitivity to MEK inhibition Nature 2006 439 358 362 16273091 10.1038/nature04304 PMC3306236

[B11] Heidorn SJ Milagre C Whittaker S Nourry A Niculescu-Duvas I Dhomen N Hussain J Reis-Filho JS Springer CJ Pritchard C Marais R Kinase-dead BRAF and oncogenic RAS cooperate to drive tumor progression through CRAF Cell 2010 140 209 221 20141835 10.1016/j.cell.2009.12.040 PMC2872605

[B12] Mouawad R Sebert M Michels J Bloch J Spano JP Khayat D Treatment for metastatic malignant melanoma: old drugs and new strategies Crit Rev Oncol Hematol 2010 74 27 39 19781957 10.1016/j.critrevonc.2009.08.005

[B13] Marchesi F Turriziani M Tortorelli G Avvisati G Torino F De Vecchis L Triazene compounds: mechanism of action and related DNA repair systems Pharmacol Res 2007 56 275 287 17897837 10.1016/j.phrs.2007.08.003

[B14] Middleton MR Grob JJ Aaronson N Fierlbeck G Tilgen W Seiter S Gore M Aamdal S Cebon J Coates A Dreno B Henz M Schadendorf D Kapp A Weiss J Fraass U Statkevich P Muller M Thatcher N Randomized phase III study of temozolomide versus dacarbazine in the treatment of patients with advanced metastatic malignant melanoma J Clin Oncol 2000 18 158 166 10623706 10.1200/JCO.2000.18.1.158

[B15] Friedman HS Kerby T Calvert H Temozolomide and treatment of malignant glioma Clin Cancer Res 2000 6 2585 2597 10914698

[B16] Hauschild A Grob JJ Demidov LV Jouary T Gutzmer R Millward M Rutkowski P Blank CU Miller WH Jr Kaempgen E Martín-Algarra S Karaszewska B Mauch C Chiarion-Sileni V Martin AM Swann S Haney P Mirakhur B Guckert ME Goodman V Chapman PB Dabrafenib in BRAF-mutated metastatic melanoma: a multicentre, open-label, phase 3 randomised controlled trial Lancet 2012 380 358 365 22735384 10.1016/S0140-6736(12)60868-X

[B17] Meckbach D Keim U Richter S Leiter U Eigentler TK Bauer J Pflugfelder A Buttner P Garbe C Weide B BRAF-V600 mutations have no prognostic impact in stage IV melanoma patients treated with monochemotherapy PLoS One 2014 9 e89218 24586605 10.1371/journal.pone.0089218 PMC3930670

[B18] Chen KG Valencia JC Gillet JP Hearing VJ Gottesman MM Involvement of ABC transporters in melanogenesis and the development of multidrug resistance of melanoma Pigment Cell Melanoma Res 2009 22 740 749 19725928 10.1111/j.1755-148X.2009.00630.x PMC2766009

[B19] Rockmann H Schadendorf D Drug resistance in human melanoma: mechanisms and therapeutic opportunities Onkologie 2003 26 581 587 14709935 10.1159/000074156

[B20] Sarasin A Dessen P DNA repair pathways and human metastatic malignant melanoma Curr Mol Med 2010 10 413 418 20455851 10.2174/156652410791317011

[B21] Verbeek B Southgate TD Gilham DE Margison GP O6-Methylguanine-DNA methyltransferase inactivation and chemotherapy Br Med Bull 2008 85 17 33 18245773 10.1093/bmb/ldm036

[B22] Bennett LL Jr Smithers D Rose LM Adamson DJ Shaddix SC Thomas HJ Metabolism and metabolic effects of 2-azahypoxanthine and 2-azaadenosine Biochem Pharmacol 1985 34 1293 1304 2859858 10.1016/0006-2952(85)90508-8

[B23] Szybalski W Use of the HPRT gene and the HAT selection technique in DNA-mediated transformation of mammalian cells: first steps toward developing hybridoma techniques and gene therapy Bioessays 1992 14 495 500 1445289 10.1002/bies.950140712

[B24] Talantov D Mazumder A Yu JX Briggs T Jiang Y Backus J Atkins D Wang Y Novel genes associated with malignant melanoma but not benign melanocytic lesions Clin Cancer Res 2005 11 7234 7242 16243793 10.1158/1078-0432.CCR-05-0683

[B25] Garnett MJ Edelman EJ Heidorn SJ Greenman CD Dastur A Lau KW Greninger P Thompson IR Luo X Soares J Liu Q Iorio F Surdez D Chen L Milano RJ Bignell GR Tam AT Davies H Stevenson JA Barthorpe S Lutz SR Kogera F Lawrence K McLaren-Douglas A Mitropoulos X Mironenko T Thi H Richardson L Zhou W Jewitt F Systematic identification of genomic markers of drug sensitivity in cancer cells Nature 2012 483 570 575 22460902 10.1038/nature11005 PMC3349233

[B26] Hoek KS Schlegel NC Brafford P Sucker A Ugurel S Kumar R Weber BL Nathanson KL Phillips DJ Herlyn M Schadendorf D Dummer R Metastatic potential of melanomas defined by specific gene expression profiles with no BRAF signature Pigment Cell Res 2006 19 290 302 16827748 10.1111/j.1600-0749.2006.00322.x

[B27] Hauser IA Renders L Radeke HH Sterzel RB Goppelt-Struebe M Mycophenolate mofetil inhibits rat and human mesangial cell proliferation by guanosine depletion Nephrol Dial Transplant 1999 14 58 63 10052478 10.1093/ndt/14.1.58

[B28] Lev DC Ruiz M Mills L McGary EC Price JE Bar-Eli M Dacarbazine causes transcriptional up-regulation of interleukin 8 and vascular endothelial growth factor in melanoma cells: a possible escape mechanism from chemotherapy Mol Cancer Ther 2003 2 753 763 12939465

[B29] Wouters J Stas M Gremeaux L Govaere O Van den Broeck A Maes H Agostinis P Roskams T van den Oord JJ Vankelecom H The human melanoma side population displays molecular and functional characteristics of enriched chemoresistance and tumorigenesis PLoS One 2013 8 e76550 24098529 10.1371/journal.pone.0076550 PMC3789681

[B30] Metelmann HR Von Hoff DD In vitro activation of dacarbazine (DTIC) for a human tumor cloning system Int J Cell Cloning 1983 1 24 32 6674387 10.1002/stem.5530010105

[B31] Shibuya H Kato Y Saito M Isobe T Tsuboi R Koga M Toyota H Mizuguchi J Induction of apoptosis and/or necrosis following exposure to antitumour agents in a melanoma cell line, probably through modulation of Bcl-2 family proteins Melanoma Res 2003 13 457 464 14512787 10.1097/00008390-200310000-00004

[B32] Lev DC Onn A Melinkova VO Miller C Stone V Ruiz M McGary EC Ananthaswamy HN Price JE Bar-Eli M Exposure of melanoma cells to dacarbazine results in enhanced tumor growth and metastasis in vivo J Clin Oncol 2004 22 2092 2100 15123733 10.1200/JCO.2004.11.070

[B33] Fiore D Jackson AJ Didolkar MS Dandu VR Simultaneous determination of dacarbazine, its photolytic degradation product, 2-azahypoxanthine, and the metabolite 5-aminoimidazole-4-carboxamide in plasma and urine by high-pressure liquid chromatography Antimicrob Agents Chemother 1985 27 977 979 4026274 10.1128/aac.27.6.977 PMC180202

[B34] Parsons PG Smellie SG Morrison LE Hayward IP Properties of human melanoma cells resistant to 5-(3′,3′-dimethyl-1-triazeno)imidazole-4-carboxamide and other methylating agents Cancer Res 1982 42 1454 1461 7060019

[B35] Saunders PP DeChang W Chao LY Mechanisms of 5-(3,3-dimethyl-1-triazeno)imidazole-4-carboxamide (Dacarbazine) cytotoxicity toward Chinese hamster ovary cells in vitro are dictated by incubation conditions Chem Biol Interact 1986 58 3 319 331 3742646 10.1016/s0009-2797(86)80106-5

[B36] Fellenberg J Kunz P Sahr H Depeweg D Overexpression of inosine 5′-monophosphate dehydrogenase type II mediates chemoresistance to human osteosarcoma cells PLoS One 2010 5 e12179 20808934 10.1371/journal.pone.0012179 PMC2922339

[B37] Penuelas S Noe V Ciudad CJ Modulation of IMPDH2, survivin, topoisomerase I and vimentin increases sensitivity to methotrexate in HT29 human colon cancer cells Febs J 2005 272 696 710 15670151 10.1111/j.1742-4658.2004.04504.x

[B38] Penuelas S Noe V Morales R Ciudad CJ Sensitization of human erythroleukemia K562 cells resistant to methotrexate by inhibiting IMPDH Med Sci Monit 2005 11 BR6 BR12 15614187

[B39] Aufderklamm S Todenhofer T Gakis G Kruck S Hennenlotter J Stenzl A Schwentner C Thymidine kinase and cancer monitoring Cancer Lett 2012 316 6 10 22068047 10.1016/j.canlet.2011.10.025

[B40] Verma S Quirt IC Eisenhauer EA Iscoe NA Young VJ Bodurtha AJ Davidson J A phase II study of weekly edatrexate (10-EDAM) in metastatic melanoma: a national cancer institute of Canada clinical trials group study Ann Oncol 1993 4 254 255 8471559 10.1093/oxfordjournals.annonc.a058467

[B41] Leahy MF Silver HK Klimo P Hall TC Treatment of advanced malignant melanoma with high dose methotrexate and folinic acid rescue Med Pediatr Oncol 1982 10 151 156 7040930 10.1002/mpo.2950100206

[B42] Chen KG Valencia JC Lai B Zhang G Paterson JK Rouzaud F Berens W Wincovitch SM Garfield SH Leapman RD Hearing VJ Gottesman MM Melanosomal sequestration of cytotoxic drugs contributes to the intractability of malignant melanomas Proc Natl Acad Sci U S A 2006 103 9903 9907 16777967 10.1073/pnas.0600213103 PMC1502551

[B43] Saez-Ayala M Fernandez-Perez MP Montenegro MF Sanchez-del-Campo L Chazarra S Pinero-Madrona A Cabezas-Herrera J Rodriguez-Lopez JN Melanoma coordinates general and cell-specific mechanisms to promote methotrexate resistance Exp Cell Res 2012 318 1146 1159 22484375 10.1016/j.yexcr.2012.03.022

[B44] Sanchez-del-Campo L Montenegro MF Cabezas-Herrera J Rodriguez-Lopez JN The critical role of alpha-folate receptor in the resistance of melanoma to methotrexate Pigment Cell Melanoma Res 2009 22 588 600 19493312 10.1111/j.1755-148X.2009.00586.x

[B45] Saez-Ayala M Montenegro MF Sanchez-Del-Campo L Fernandez-Perez MP Chazarra S Freter R Middleton M Pinero-Madrona A Cabezas-Herrera J Goding CR Rodriguez-Lopez JN Directed phenotype switching as an effective antimelanoma strategy Cancer Cell 2013 24 105 119 23792190 10.1016/j.ccr.2013.05.009

[B46] Robien K Boynton A Ulrich CM Pharmacogenetics of folate-related drug targets in cancer treatment Pharmacogenomics 2005 6 673 689 16207145 10.2217/14622416.6.7.673

[B47] Watson AJ Margison GP O (6)-alkylguanine-DNA alkyltransferase assay Methods Mol Med 1999 28 167 178 21374037 10.1385/1-59259-687-8:167

[B48] Gerson SL Trey JE Miller K Berger NA Comparison of O6-alkylguanine-DNA alkyltransferase activity based on cellular DNA content in human, rat and mouse tissues Carcinogenesis 1986 7 745 749 3698202 10.1093/carcin/7.5.745

[B49] Watson AJ Margison GP O6-alkylguanine-DNA alkyltransferase assay Methods Mol Biol 2000 152 49 61 10957968 10.1385/1-59259-068-3:49

[B50] Elder RH Margison GP Rafferty JA Differential inactivation of mammalian and Escherichia coli O6-alkylguanine-DNA alkyltransferases by O6-benzylguanine Biochem J 1994 298 Pt 1 231 235 8129725 10.1042/bj2980231 PMC1138006

