# Supplementary material for: Differential chemosensitivity to antifolate drugs between RAS and BRAF melanoma cells
Source: Mol Cancer. 2014 Jun 19;13:154. doi: 10.1186/1476-4598-13-154 (PMC4079649; doi:10.1186/1476-4598-13-154)
Supplement: Additional file 1: Table S1 — List of melanoma cell lines used in this study, grouped upon the presence of activating mutations in either BRAF or RAS. [file 1476-4598-13-154-S1.pdf]

Cell line	BRAF mutation
A375P	V600E
WM164	V600E
D10	V600E
Skmel28	V600E
WM266.4	V600D
WM9	V600E
WM98.1	V600E
WM1158	V600E
501mel	V600E

Cell line	RAS mutation
Skeml2	KRAS Q61R
D04	NRAS Q61L
WM852	NRAS Q61R
MM415	NRAS Q61L
MM485	NRAS Q61R
MelJuso	NRAS Q61L/ HRAS G13D
WM1361	NRAS Q61K
CJM	NRAS Q61K
WM1366	NRAS Q61L

Supplementary Table 1. List of melanoma cell lines used in this study, grouped upon the presence of activating mutations in either BRAF or RAS.
