# Supplementary material for: Differential chemosensitivity to antifolate drugs between RAS and BRAF melanoma cells
Source: Mol Cancer. 2014 Jun 19;13:154. doi: 10.1186/1476-4598-13-154 (PMC4079649; doi:10.1186/1476-4598-13-154)
Supplement: Additional file 2: Figure S1 — A, DTIC exposure to white light increases DTIC’ inhibitory effect. [file 1476-4598-13-154-S2.pdf]

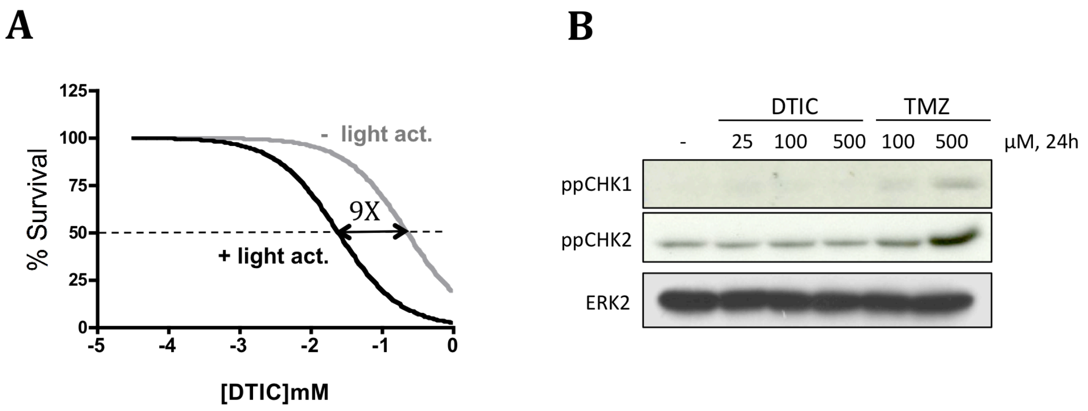

Supplementary Figure 1.

A, DTIC exposure to white light increases DTIC' inhibitory effect. Dose response curve of WM266-4 cell survival to light activated (+ light act.) or non-activated (- light act.) DTIC. Untreated cells were set as 100%. Light exposure reduced the IC50 for DTIC 9-fold. **B, Light activated DTIC doesn't activate CHK-kinases.** A375P cells were treated for 24h with DTIC or TMZ as indicated. Cell lysates were prepared as described (Wellbrock et al., 2008) and analysed by standard Western-blotting protocols for the expression of active CHK1 and 2. Antibodies used were: anti-phosphoCHK1 (ser345), anti-phosphoCHK2 (Thr68) from Cell Signalling and, as a loading control, anti-ERK2 (C-14) from Santa Cruz Biotechnology, Santa Cruz, CA, USA.
