# Supplementary material for: Differential chemosensitivity to antifolate drugs between RAS and BRAF melanoma cells
Source: Mol Cancer. 2014 Jun 19;13:154. doi: 10.1186/1476-4598-13-154 (PMC4079649; doi:10.1186/1476-4598-13-154)
Supplement: Additional file 3: Table S2 — MGMT status of a panel of 10 melanoma cell lines MGMT activity in 5 mutBRAF and 5 mutNRAS melanoma cell lines. Exponentially growing cells were harvested, gDNA was isolated and the presence of MGMT activity as well as the concentration of MGMT was determined. N.d.: not detectable. [file 1476-4598-13-154-S3.pdf]

Cell line	GENETIC BACKGROUND	MGMT fmol/µg DNA	MGMT molecules/cell	MGMT STATUS
**Mel-juso**	mutNRAS	n.d.	~0	deficient
**WM1361**	mutNRAS	4.4	~8000	proficient
**MM485**	mutNRAS	n.d.	~0	deficient
**WM852**	mutNRAS	n.d.	~0	deficient
**SKMEL2**	mutKRAS	12.8	~23300	proficient
**D10**	mutBRAF	13.1	~23800	proficient
**WM266.4**	mutBRAF	n.d.	~0	deficient
**501MEL**	mutBRAF	6.6	~12000	proficient
**WM9**	mutBRAF	n.d.	~0	deficient
**WM98**	mutBRAF	n.d.	~0	deficient
**A375P**	mutBRAF	5.6	~10200	proficient
**MCF7**	(positive control)	8.8	~16000	proficient

Supplementary Table 2. MGMT status of a panel of 10 melanoma cell lines

MGMT activity in 5 mutBRAF and 5 mutNRAS melanoma cell lines. Exponentially growing cells were harvested, gDNA was isolated and the presence of MGMT activity as well as the concentration of MGMT was determined. N.d.: not detectable.
