# Supplementary material for: Differential chemosensitivity to antifolate drugs between RAS and BRAF melanoma cells
Source: Mol Cancer. 2014 Jun 19;13:154. doi: 10.1186/1476-4598-13-154 (PMC4079649; doi:10.1186/1476-4598-13-154)
Supplement: Additional file 4: Figure S2 — 2-Azahypoxanthine and light activated DTIC show similar UV absorbance profiles. [file 1476-4598-13-154-S4.pdf]

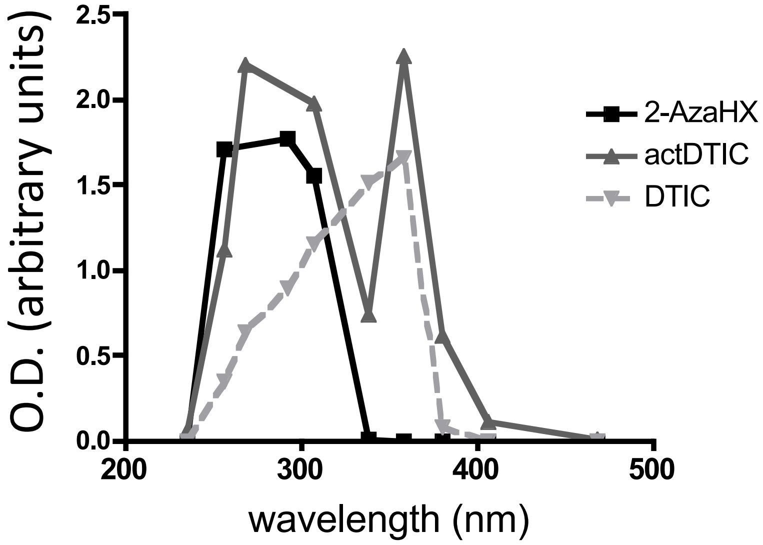


**Supplemental Figure 2. 2-Azahypoxanthine and light activated DTIC show similar UV absorbance profiles.** The absorption profiles for 0.05mM 2-AzaHypoxanthine and 0.1mM light activated DTIC were obtained using an ultra low volume spectro-photometer (NanodropND1000, Lab Tech). Light exposed DTIC shows a UV absorption profile (dark-gray line) with two peaks of maximal absorption. One at 350-370nm (overlapping the UV spectrum for non activated DTIC, dashed light-gray line) and one at 256-295nm overlapping the UV absorption profile corresponding to 2-Azahypoxanthine (black line).
